# Editors, Publishers, Impact Factors, and Reprint Income

**DOI:** 10.1371/journal.pmed.1000355

**Published:** 2010-10-26

**Authors:** Harvey Marcovitch

**Affiliations:** Clinical Risk, RSM Press Ltd., London, United Kingdom

## Abstract

Harvey Marcovitch discusses new research findings from Andreas Lundh and colleagues that examined the effect of publishing industry-funded clinical trials on journal citations and reprint income at six major medical journals.

## Editors under Pressure—Avoiding Conflicts

Editors would like to imagine they are simply gatekeepers who facilitate the interaction between authors who wish to impart information and people who want to read it. In fact, they are subject to a raft of external pressures that interfere with this core task. Coauthors are prone to disputes with each other and with reviewers; rejected authors may protest; readers may be dissatisfied; institutions may react inadequately to editors' concerns about probity; editorial freedom may be compromised by the demands of the learned society that owns the journal; and a commercial publisher might exert subtle—or unsubtle—pressure to increase profitability. All of these distractions increase the possibility of competing interests corrupting the editorial process.

## Influence of the Impact Factor

Added to this toxic mixture is the impact factor (IF). Just as many clinicians claim that contacts by pharmaceutical company representatives do not affect their prescribing behaviour, so editors are likely to deny that thoughts of a rising IF might influence their acceptance rates. In their paper published this week in *PLoS Medicine*, Andreas Lundh and colleagues analysed randomised controlled trials published in six high-impact general medical journals during two time periods a decade apart; they calculated the putative fall in IF that would have occurred had publication been denied to papers that were commercially sponsored [1].

Unsurprisingly, they found the expected association, since IF depends in turn on recent citation rates, and a body of literature shows that industry-sponsored trials attract more citations than those funded by a nonprofit source [2],[3]. There are reasons: for example, randomised controlled trials and meta-analyses are cited more frequently than clinical studies with less-rigorous design, regardless of funding, and high-impact journals are likely to attract the former [4]. There is “gamesmanship,” with commercial sponsors and publishing companies skilled at obtaining publicity in the mass media, a known stimulant for citations [5],[6]. Only slightly dubious is the habit of commercial companies disseminating papers favourable to their product to individual clinicians at conferences or through sponsorship of review papers—themselves a potent accelerator of IF [7]. More culpable is the fact that studies showing positive outcomes for a drug or device under consideration are more likely to be published than “negative” studies; editors are partly to blame for this but so are commercial sponsors, whose methodologically well-conducted studies with unfavourable results tended not to see the light of day, at least in the pretrial registration era [8].

Lundh and colleagues do not claim that their findings demonstrate that editors' judgment on acceptance or rejection is influenced by the paper's predicted effect on IF. Nonetheless, I have heard editors support a paper at a selection meeting by stating that it is likely to be well cited.

## Publishers' Profits and Sponsored Studies

More intriguingly, the second aim of their study was to investigate the possible financial benefits to publishers of the journals they investigated. Their attempted method was to seek data on income from advertisements, reprints, and industry-supported supplements as a percentage of total income. The editors of the two UK-based journals, *BMJ* and *The Lancet*, provided the data. The editors of *JAMA* and *The New England Journal of Medicine (NEJM)* declined, as did the publisher of *Annals of Internal Medicine*. The owners of the latter confirmed the proxy data obtained from the US Internal Revenue Service but the publishers of the former two journals, the American Medical Association and the Massachusetts Medical Society, did not respond.

Again, the authors cannot infer the intentions of their nonrespondent editors. Editors are proud of their independence but independence goes only as far as an owner permits, as we know from the sorry history of dismissed editors of *NEJM* and the *Canadian Medical Association Journal*, amongst others.

## Journals as Leaders: A Paradox

In many ways, JAMA has led the way in promoting publishing integrity—including its call for independent statistical analysis of submitted industry-sponsored trials [9] and its publication of best practice recommendations for professional medical associations [10]. The authors of the latter paper, who include the journal's editor-in-chief, state: “Professional medical associations have a duty to bring to their members the best scientific evidence on the efficacy and suitability of drugs and devices. These efforts must be separate from and not affected by industry promotions.” It is a paradox that the professional medical association that owns JAMA was less than open and transparent with Lundh and colleagues about potential financial conflicts (such as their income from industry sources) as they expect their authors to be.

## Stronger Guidance Needed

The various bodies that advise editors may need to strengthen their guidance. The Council of Science Editors' White Paper on Promoting Integrity in Scientific Journal Publication [11] includes as a potential conflict for editors “employment by an organisation that would obtain some advantage from a favourable product-related publication.” The International Committee of Medical Journal Editors (ICMJE) states that editors who make the final decisions about manuscripts must have no “personal, professional or financial involvement in any of the issues they might judge.” The Committee on Publication Ethics (COPE) code of conduct for editors requires them to prevent business needs from compromising important intellectual standards. None of these organisations comment on the potential conflicts that might arise when a journal or publisher receives a substantial proportion of its income from reprints (23%, Massachusetts Medical Society; 41%, *The Lancet*; 53%, American Medical Association).

Journal editors have expended much time and effort in teasing out how to handle authors' and reviewers' competing interests. They need now to concentrate on their own and those of their employers, lest we reach the dismal scenario described by Marcia Angell: “it is simply no longer possible to believe much of the clinical research that is published, or to rely on the judgment of trusted physicians or authoritative medical guidelines. I take no pleasure in this conclusion, which I reached slowly and reluctantly over my two decades as an editor of *The New England Journal of Medicine*” [12].

## Abbreviations

IF: impact factor
